# *BolANT3* Positively Regulates Indolic Glucosinolate Accumulation by Transcriptionally Activating *BolCYP83B1* in Cabbage

**DOI:** 10.3390/ijms26073415

**Published:** 2025-04-05

**Authors:** Chengtai Yan, Wenjing Yang, Xuemei Yan, Yao Liu, Jiahao Zhang, Xue Bai, Qi Zeng, Xifan Liu, Dengkui Shao, Baohua Li

**Affiliations:** 1 State Key Laboratory for Crop Stress Resistance and High-Efficiency Production, College of Horticulture, Northwest A & F University, Yangling 712100, China; yan1618226@nwafu.edu.cn (C.Y.); yangwenjing@nwafu.edu.cn (W.Y.); yanxuemei@nwafu.edu.cn (X.Y.); lliuyao@nwafu.edu.cn (Y.L.); zjh_nwafu@nwafu.edu.cn (J.Z.); xue.bai@nwafu.edu.cn (X.B.); zengqi@nwafu.edu.cn (Q.Z.); liuxifan2020051572@nwafu.edu.cn (X.L.); 2 Academy of Agriculture and Forestry Sciences, Qinghai University, Xining 810016, China; 3 Qinghai Key Laboratory of Vegetable Genetics and Physiology, Xining 810016, China

**Keywords:** cabbage, indolic glucosinolates, *BolCYP83B1*, *BolANT3*, transcriptional regulation

## Abstract

Indolic glucosinolates are a group of plant secondary metabolites found in *Brassica* vegetables, and their breakdown products could act as important anti-cancer and defense compounds against biotic stresses. Transcriptional regulation plays a key role in modulating the biosynthesis of indolic glucosinolates in the model plant *Arabidopsis*, but little is known about the transcriptional regulatory landscape of these glucosinolates in *Brassica* vegetables. In this study, we selected and functionally validated the important biosynthetic gene *BolCYP83B1* from the indolic glucosinolate pathway in cabbage. Through a yeast one-hybrid assay, we systemically screened and identified upstream regulators of *BolCYP83B1* in cabbage with BolANTs as the top candidates for further functional validation. Two homologs of BolANTs, BolANT1 and *BolANT3*, were confirmed to bind the promoter of *BolCYP83B1* via both a yeast one-hybrid assay and an LUC assay. The overexpression of *BolANT3* in cabbage significantly increased the accumulation of indolic glucosinolates, while the virus-induced gene silencing (VIGS) of *BolANT3* significantly reduced the accumulation of indolic glucosinolates in cabbage. Our work provides valuable insights into the transcriptional regulatory mechanisms of indolic glucosinolates in *Brassica* vegetables.

## 1. Introduction

Plants provide raw materials for Earth’s ecosystem and for the survival of human society mainly through large amounts of plant metabolites, which can be generally classified as plant primary metabolites, plant secondary metabolites, and plant hormones [1]. Metabolites with an indole ring are of particular interest and belong to all three categories, including tryptophan as a plant primary metabolite, glucosinolates as plant secondary metabolites, and auxin as a plant hormone. Conducting research to understand the biological functions and the regulation of the accumulation of metabolites with indole rings remains one of the frontiers in plant biology.

Indolic glucosinolates are a group of plant secondary metabolites with tryptophan as their biosynthetic precursor and are mostly present in the order of *Brassicales*, including the model plant *Arabidopsis* and the economically important *Brassica* vegetables [2,3]. Indolic glucosinolates play key roles in plant defense responses [4,5] and serve as key functional nutrients in *Brassica* vegetables [6,7]. As glucosinolates are energy-intensive compounds and utilize approximately 15% of all plants’ energy [8], the biosynthesis of glucosinolates, including indolic glucosinolates, is under tight regulation. Transcriptional regulation is the most important regulatory tool in modulating plant secondary metabolites according to current research, and key regulators in multiple plant species with the power to turn the whole pathway on and off were identified recently, including *Bl* (*Bitter leaf*) and *Bt* (*Bitter fruit*) in the biosynthesis of cucurbitacin C in cucumber [9] and *LATE MERISTEM IDENTITY1* (*LMI1*) in the biosynthesis of essential oil in citrus [10]. Key transcriptional regulators of indolic glucosinolates, MYB34, MYB51, and MYB122, were also identified and functionally validated, but indolic glucosinolates were not detected in the *myb34myb51myb122* triple mutant [11,12,13,14]. The high potential of the transcriptional regulation of indolic glucosinolates has attracted increasing attention for translational applications and breeding projects.

*Brassica* vegetables play important roles in providing rich nutrients to billions of people around the world, especially those in underdeveloped regions. Indol-3-ylmethyl (I3M) is one of the key functional nutrients in *Brassica* vegetables, and a breakdown product of I3M, indole-3-carbinol (I3C), was found to be an active and important functional nutrient with significant anti-cancer activities [6,7,15]. Also important is 4-methoxy-indol-3-ylmethyl (4MOI3M) from indolic glucosinolates, which plays a key role in plants’ defense responses to biotic stresses in both *Brassica* vegetables and *Arabidopsis* [4,5,16,17]. All of these findings further support the utilization of indolic glucosinolates in translational applications as key nutrients and green pesticides, and transcriptional regulation is one of the most powerful ways to fulfill these goals.

Considering the important and diverse functions of indolic glucosinolates, we explored the transcriptional regulation of their biosynthesis in cabbage by screening and validating the upstream regulators of *BolCYP83B1* for the following reasons: (1) BolCYP83B1 catalyzes the key step in the core structure formation of the biosynthesis of indolic glucosinolates [18,19]; (2) *CYP83B1* is the gene with one of the largest numbers of upstream candidate regulators in the model plant *Arabidopsis* according to our previous study [20,21,22]; and (3) the expression level of *BolCYP83B1* has profound influences on the accumulation of the key plant hormone auxin, therefore modulating plant development [23], which gives us the opportunity to study how plants coordinate defense and development. The objectives of this study were to identify and functionally validate the *BolCYP83B1* of cabbage, screen its upstream regulators, and functionally validate one of its top candidate regulators, *BolANT3*, in cabbage overexpression lines and virus-induced gene silencing (VIGS) lines. The functional validation of *BolANT3*, together with the other candidate regulators of *BolCYP83B1,* provides valuable insights into how indolic glucosinolates are transcriptionally regulated, making them prime targets for breeding new cabbage cultivars with higher nutritional value and enhanced defense responses.

## 2. Results

### 2.1. Transcriptional Regulation of CYP83B1 in Arabidopsis

**Figure 1:**
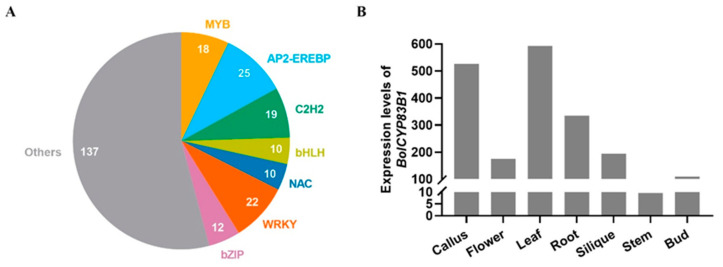
Expression and transcriptional regulation of *CYP83B*. (**A**) Distribution of upstream candidate regulatory transcription factors of *AtCYP83B1.* (**B**) Expression of *BolCYP83B1* in representative tissues of cabbage.

As the key biosynthetic enzyme converting indole-3-acetaldoxime (IAOx) into the following core structural formation steps of the indolic glucosinolate pathway, CYP83B1 not only plays an important role in the biosynthesis of indolic glucosinolates but also modulates the accumulation of camalexin and the key plant hormone auxin, all of which are downstream metabolic products of IAOx [18,23,24]. Therefore, *CYP83B1* was selected as one of several important control genes in our efforts over the past 15 years to explore the transcriptional regulation of the aliphatic glucosinolate pathway using an enhanced yeast one-hybrid assay [20,21,22]. In these studies, we identified 253 transcription factors (TFs) that bind to the promoter of *CYP83B1* in *Arabidopsis* (Supplementary Table S1), and interestingly, the numbers of upstream regulators of *CYP83B1* in our screening are some of the highest among all of the tested promoters [22]. These upstream regulators of *CYP83B1* belong to multiple TF families, including MYB, AP2-EREBP, C2H2, bHLH, NAC, WRKY, and bZIP (Figure 1A), supporting their important and diverse biological roles in modulating key metabolites in primary metabolites, secondary metabolites, and plant hormones.

### 2.2. Functional Validation of BolCYP83B1 in Cabbage

We used cabbage to study the transcriptional regulation of glucosinolates and investigated the underlying regulatory mechanisms over the past 5 years [25,26]. In this study, we continued to explore how *CYP83B1* is transcriptionally regulated to influence the biosynthesis of indolic glucosinolates in cabbage. Although genomic polyploidization occurred in cabbage [27], only one *CYP83B1* gene (*Bol033477*) was identified in the genome, and it was renamed *BolCYP83B1* and used in the following experiment. *BolCYP83B1* showed the highest expression in leaf, supporting its role in the biosynthesis of indolic glucosinolates and its other role in modulating the accumulation of plant defense compounds against herbivores [8].

**Figure 2:**
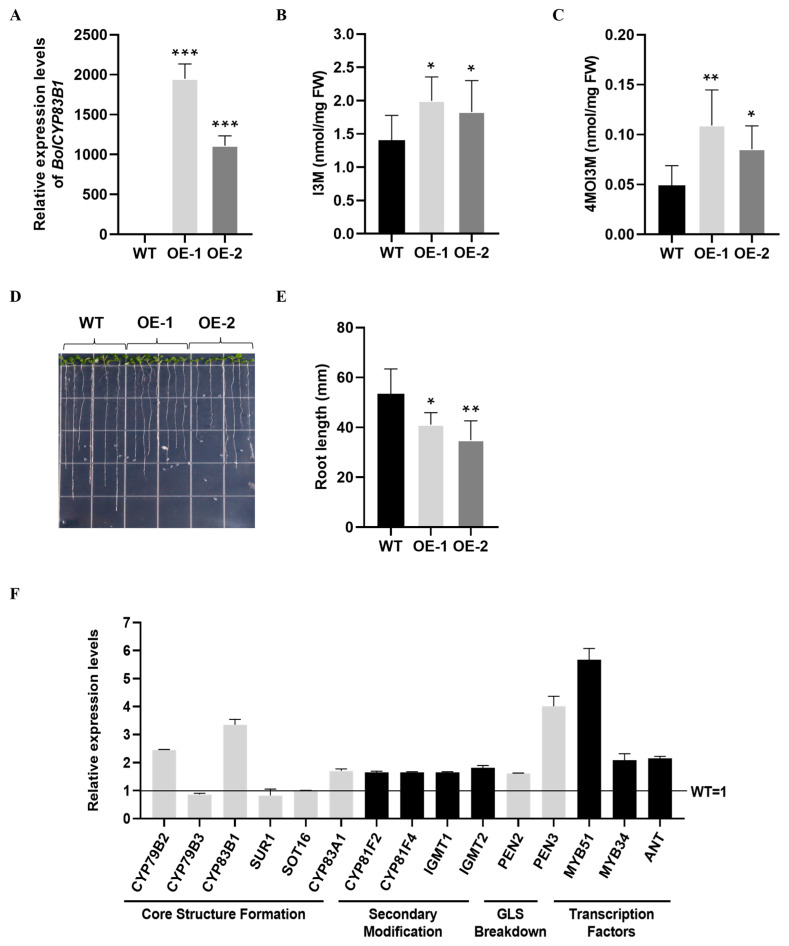
Functional validation of *BolCYP83B1.* (**A**) Expression of *BolCYP83B1* in transgenic overexpression lines of *Arabidopsis*. (**B**) I3M content in *BolCYP83B1* overexpression lines of *Arabidopsis*. (**C**) 4MOI3M content in *BolCYP83B1* overexpression lines of *Arabidopsis*. (**D**,**E**) Root length of *BolCYP83B1* in overexpression lines of *Arabidopsis*. (**F**) Expression of representative genes involved in indolic glucosinolates pathway in *BolCYP83B1* overexpression lines of *Arabidopsis*. *: *p* < 0.05; **: *p* < 0.01; ***: *p* < 0.001.

To experimentally validate the biological functions of *BolCYP83B1*, we overexpressed *BolCYP83B1* in the Col-0 ecotype of *Arabidopsis* (Figure 2A). Both I3M and 4MOI3M were significantly induced in these two independent OE lines of *BolCYP83B1* (Figure 2B,C, Supplementary Table S2). Furthermore, the root lengths of both OE lines of *BolCYP83B1* were also significantly reduced (Figure 2D,E), supporting *BolCYP83B1*’s important role in plant development by potentially modulating auxin biosynthesis. The expression of multiple biosynthetic genes in the indolic glucosinolate pathway was also induced by the overexpression of *BolCYP83B1*. Interestingly, the key regulatory gene of the indolic glucosinolate pathway, *MYB51*, was also highly induced (Figure 2F), indicating the complex and dynamic nature of the regulatory networks of indolic glucosinolates.

### 2.3. Screening of Upstream Regulators of BolCYP83B1

**Figure 3:**
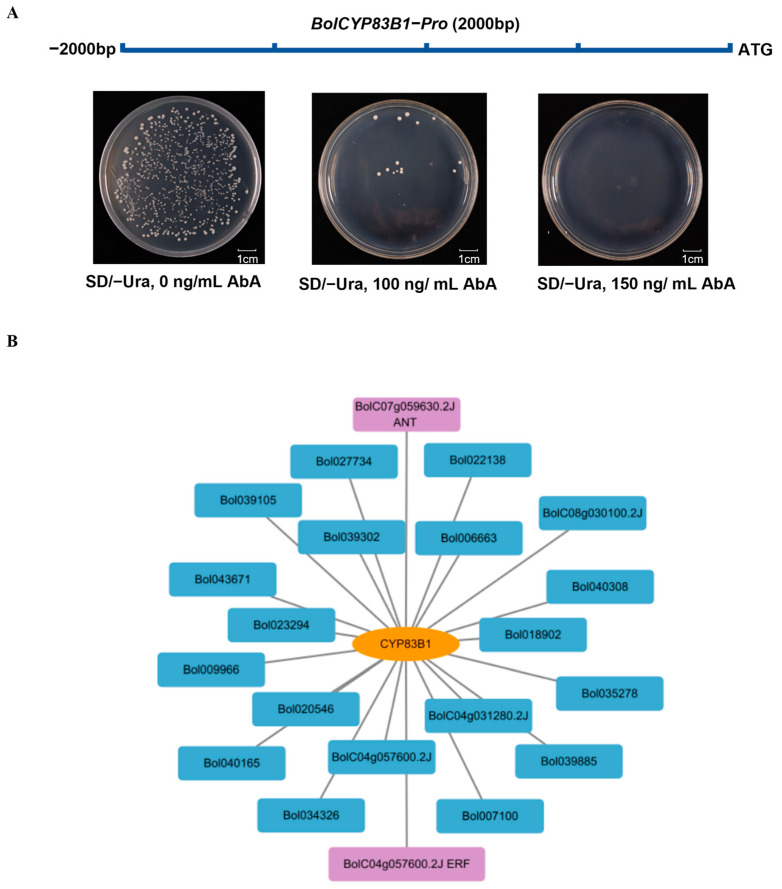
The yeast one-hybrid screening of upstream regulators of *BolCYP83B1*. (**A**) The auto activation test of the *BolCYP83B1* promoter. (**B**) The candidate regulators of *BolCYP83B1* visualized using Cytoscape(v3.9.1).

After the validation of *BolCYP83B1*’s biological roles in the indolic glucosinolate biosynthetic pathway, we studied the transcriptional regulators of *BolCYP83B1*. Firstly, we explored the cis-elements of the promoters of *CYP83B1* genes from both *Arabidopsis* and cabbage and found that their promoters contained many cis-elements in the 2 kb regions upstream of the start codon, including MYB binding sites, wound-responsive elements, and auxin responsive elements. These findings further support the biological roles of *CYP83B1* as an integrated point in *Brassica* plants’ metabolic networks to coordinate the biosynthesis of plant secondary metabolites, phytoalexin, and plant hormones (Supplemental Figure S1). The 2 kb promoter region upstream of the start codon of *BolCYP83B1* was cloned, tested, and confirmed for its low auto-activation, and it could be used for yeast one-hybrid screening (Figure 3A). By using the cDNA library prepared by our lab to screen the upstream regulator of *BolCYP83B1*, we identified 21 of the candidate upstream regulators (Figure 3B, Supplementary Table S3). Notably, *BolCYP83B1* was the first biosynthetic gene in the indolic glucosinolate pathway whose upstream regulators were screened and identified via yeast one-hybrid assays in cabbage. The candidate upstream regulators in this current study, together with the ones identified in *Arabidopsis*, serve as valuable resources and clues for understanding the transcriptional regulation of indolic glucosinolate biosynthesis in *Brassica* vegetables.

### 2.4. BolANT1 and BolANT3 Bind the Promoter of BolCYP83B1

By studying the upstream regulators of *BolCYP83B1*, we showed that AINTEGUMENTA (ANT) and ERF are transcriptional factors and top candidates for functional validation. Interestingly, BolANTs were independently identified twice in the yeast one-hybrid screening and are one of the top confirmed regulators of the aliphatic glucosinolate pathway, modulating both glucosinolate accumulation and plant growth [22]. Therefore, we selected the transcription factor ANT as the top candidate for the functional validation of its novel role in regulating indolic glucosinolate biosynthesis.

**Figure 4:**
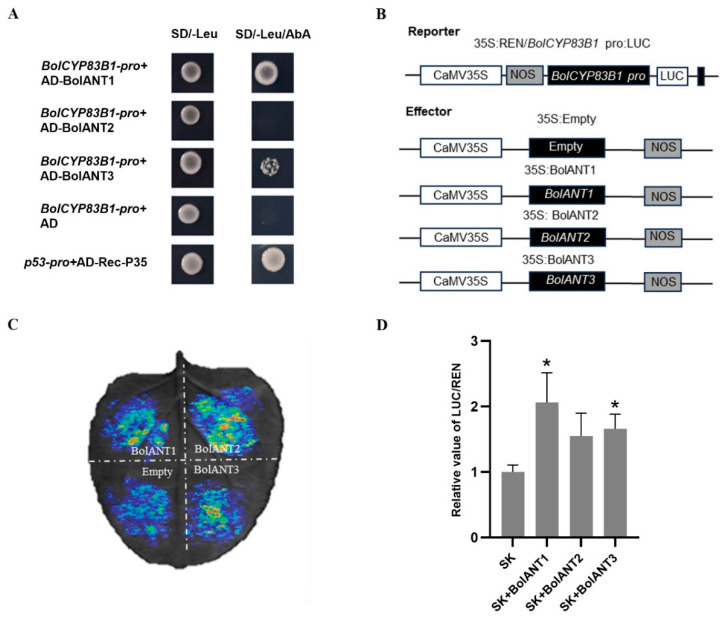
BolANT1 and *BolANT3* bind the promoters of *BolCYP83B*. (**A**) BolANT1 and *BolANT3* bind the promoter of *BolCYP83B1,* double confirmed by a yeast one-hybrid assay. (**B**) Constructions of a dual-luciferase reporter system (LUC) for BolANTs and the promoter of *BolCYP83B1*. (**C**,**D**) BolANT1 and *BolANT3* bind the promoter of *BolCYP83B1*, confirmed by an LUC assay. *: *p* < 0.05.

**Figure 5:**
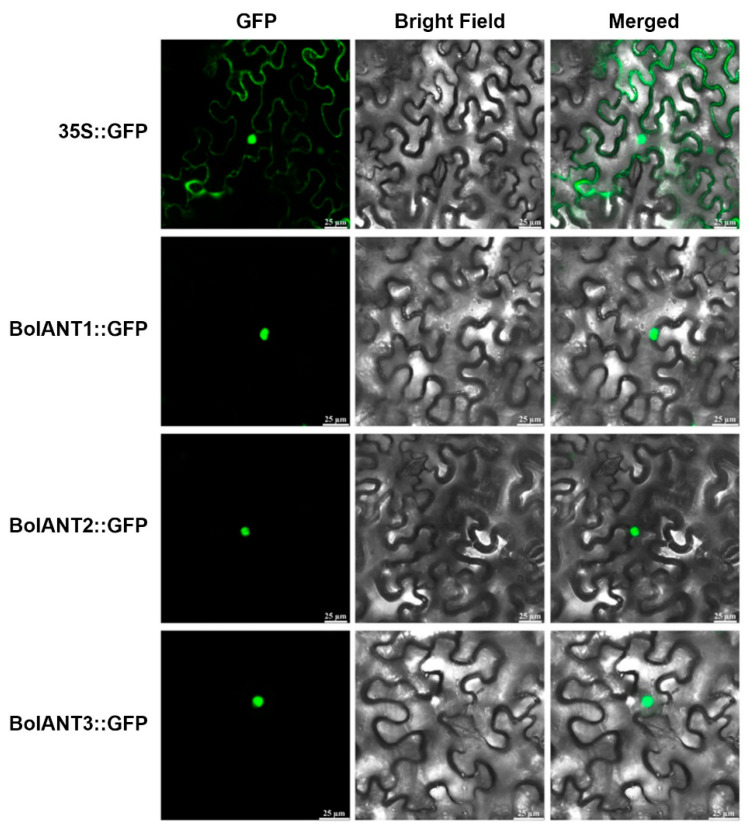
Subcellular localization analysis of BolANT1, BolANT2, and *BolANT3* proteins. Scale bar = 25 μm. The BolANTs-GFP fusion protein were located in the nucleus.

We blasted the genomic data of cabbage (http://brassicadb.cn, accessed on 10 December 2021) and identified three homologs of *ANT* and renamed them *BolANT1* (*BolC01g001400.2J*), *BolANT2* (*BolC07g059630.2J*), and *BolANT3* (*BolC03g073740.2J*). *BolANT1*, *BolANT2*, and *BolANT3* are highly homologous to *Bra017852*, *Bra011782*, and *Bra010610* in Chinese cabbage, respectively (Supplemental Figure S2). By using the yeast one-hybrid assay (Figure 4A) and the LUC assay (Figure 4B–D), we demonstrated that BolANT1 and *BolANT3* can bind the promoter of *BolCYP83B1*. The subcellular localizations of BolANT1, BolANT2, and *BolANT3* were all shown to be located in the nucleus (Figure 5), supporting their biological roles as transcriptional regulators.

### 2.5. Overexpression of BolANT3 Induced Indolic Glucosinolate Accumulation in Cabbage

To validate the biological functions of *BolANTs*, we overexpressed *BolANT1*, *BolANT2*, and *BolANT3* in cabbage and successfully obtained two stable transformation lines of *BolANT3*. Therefore, we focused on validating *BolANT3*’s biological function in this work.

**Figure 6:**
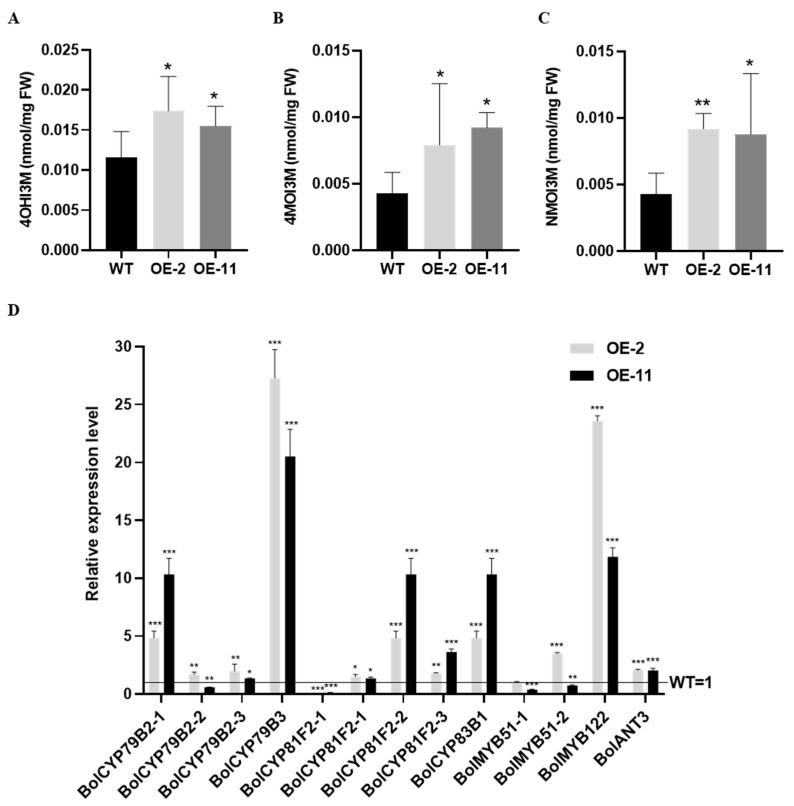
Overexpression of *BolANT3* increased indolic glucosinolate content in cabbage. (**A**) 4OHI3M content of overexpression lines of *BolANT3* in cabbage. (**B**) 4MOI3M content of overexpression lines of *BolANT3* in cabbage. (**C**) NMOI3M content of overexpression lines of *BolANT3* in cabbage. (**D**) Expression of representative genes in glucosinolates pathway in overexpression lines of *BolANT3* in cabbage. OE-2 and OE-11 are two overexpression lines of *BoANT3* *: *p* < 0.05; **: *p* < 0.01; ***: *p* < 0.001.

In the two independent lines of *BolANT3*, three major indolic glucosinolates, 4-hydroxy glucobrassicin (4OHI3M), 4-methoxyindol-3-ylmethyl glucosinolate (4MOI3M), and *N*-methoxy-indol-3-ylmethyl glucosinolate (NMOI3M), were all significantly induced, supporting *BolANT3*’s role as a positive regulator of indolic glucosinolates (Figure 6A–C). The qPCR analysis showed that the overexpression of *BolANT3* significantly increased the expression of *BolCYP83B1*, and interestingly, the key regulator of the indolic glucosinolate pathway, *MYB122*, was also highly induced in the overexpression lines of *BolANT3* (Figure 6D), indicating potential hierarchical regulatory roles of BolANTs in indolic glucosinolate biosynthesis.

### 2.6. VIGS of BolANT3 Repressed Indolic Glucosinolate Accumulation in Cabbage

**Figure 7:**
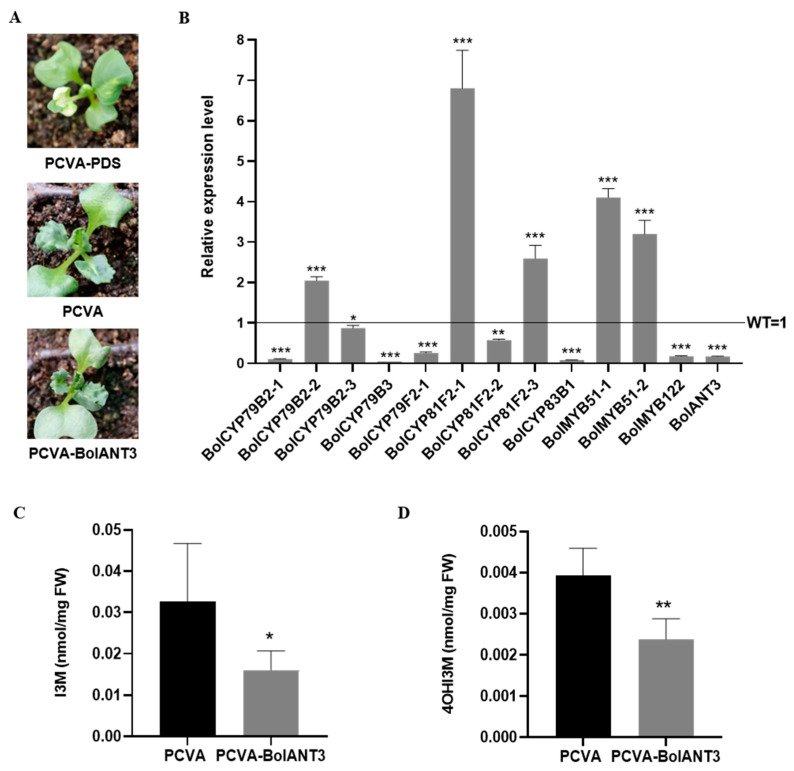
The silencing of *BolANT3* reduced the indolic glucosinolate content in cabbage. (**A**) The phenotypes of the *BolANT3*-silenced lines in cabbage obtained via a VIGS assay. (**B**) The expression of representative genes in the indolic glucosinolate pathway in *BolANT3*-silenced lines in cabbage. (**C**) The I3M content of *BolANT3*-silenced lines in cabbage. (**D**) The 4OHI3M content of *BolANT3*-silenced lines in cabbage. *: *p* < 0.05; **: *p* < 0.01; ***: *p* < 0.001.

To further confirm the biological role of *BolANT3*, we used VIGS to repress the expression of *BolANT3*, and the positive control of silencing phytoene desaturase (PDS) showed a clear white leaf, indicating that the assay was functional (Figure 7A). The expression of *BolANT3* was significantly reduced compared with the one in the control, and accordingly, the expression of *BolCYP83B1* was also significantly reduced. This confirms that *BolANT3* positively regulates the expression of *BolCYP83B1*. Accordingly, the accumulation of major indolic glucosinolates, I3M and 4OHI3M, was significantly lower than in the control lines. The VIGS assay in cabbage, together with the overexpression lines in cabbage, strongly indicate that *BolANT3* is a positive regulator of *BolCYP83B1* and may induce the accumulation of indolic glucosinolates in cabbage.

## 3. Discussion

### 3.1. Value of Transcriptional Regulation of Indolic Glucosinolates in Brassica Vegetables

Manipulating the biosynthesis of plant secondary metabolites is one of the most important research areas in plant biology [28,29]. Until now, transcriptional regulation has been found to be the most important regulatory approach for plants’ control of the biosynthesis of secondary metabolites [9,10,11,20,30,31,32,33]. Largely because *Arabidopsis* serves as a model plant with large amounts of genetic resources and genomic data [34,35,36,37], glucosinolates eventually evolved as one of the most intensively studied systems for exploring the biology of plant secondary metabolites. Over the past 15 years, many studies have been conducted to study the transcriptional regulation of the aliphatic glucosinolate pathway, with dozens of upstream regulators being identified and validated [20,21,22,38,39,40]. In this study, we selected the *BolCYP83B1* gene from the indolic glucosinolate pathway, identified its upstream regulators, and validated one of its top candidates using a yeast one-hybrid assay. This work provides valuable information for understanding the transcriptional regulation of indolic glucosinolates, as well as insights into the conserved and diverse regulatory mechanisms between *Arabidopsis* and domesticated *Brassica* vegetables [41]. Moreover, the successful validation of *BolANT3* as an upstream regulator of *BolCYP83B1*, together with the promising phenotypes of the transgenic cabbage lines in this study, demonstrate the high potential and value of exploring the transcriptional regulatory landscape of indolic glucosinolate biosynthesis by screening more key genes in the indolic glucosinolate pathway. The candidate upstream regulators identified in our current study could also serve as ‘baits’ in co-expression analysis to help identify additional novel candidate regulators of indolic glucosinolate biosynthesis.

### 3.2. The Transcriptional Regulation of Plant Defense and Development by the BolANT3-BolCYP83B1 Module

Glucosinolates serve as major defense compounds in *Arabidopsis* and *Brassica* vegetables and play key roles in defending against herbivores and fungal and bacterial infections. As a large proportion of plants’ total energy was estimated to make glucosinolates [8], the coordination of the dynamic accumulation of glucosinolates is not only important for plants’ defense responses but also influences the allocating valuable energy between plant defense and plant development. Therefore, we selected *BolCYP83B1* from the indolic glucosinolate pathway as the target gene in this study. Although *BolCYP83B1* is the biosynthetic gene involved in the second step of the indolic glucosinolate pathway, it is a committed step for this pathway. Meanwhile, the expression and activity of *BolCYP83B1* significantly influence auxin biosynthesis, thus influencing plant development [23]. The identification and functional validation of upstream regulators of *BolCYP83B1* may potentially help develop an understanding of how plants coordinate plant development and plant defense, which was a significant research area over the past decade [22]. Supporting our hypothesis, the top candidate upstream regulator of *BolCYP83B1* is the ANT transcriptional factor, which was previously identified as a key regulator controlling the development of plant reproductive organs in *Arabidopsis* [42,43,44]. In this study, we independently identified ANT twice in the yeast one-hybrid assay, and the functional validation found that *BolANT3* is an important and positive regulator of indolic glucosinolates according to its overexpression lines and VIGS lines. It would be interesting to investigate whether the overexpression of *BolANT3* could influence plants’ reproductive development. The further exploration of BolANTs’ developmental phenotypes would help us to understand how plants coordinate plant growth and defenses through the newly identified BolANTs-*BolCYP83B1* module.

### 3.3. Disease Resistance, Nutrition Value, and Better Growth Phenotypes Achieved by Manipulating the Upstream Regulators of BolCYP83B1

As *BolCYP83B1* serves as a key biosynthetic gene in the complex metabolic networks controlling *Brassica* vegetables’ defense, nutrients, and plant hormone, the upstream regulators of *BolCYP83B1* in our current work would likely show high potential as target genes for breeding new cultivars with enhanced disease resistance, higher nutrition value, and better growth phenotypes. Through the overexpression of *BolANT3* in cabbage, *BolANT3* was shown to significantly increase the accumulation of 4MOI3M, which plays key roles in plant defense responses to biotic stresses [4,5,16]. It will be interesting to explore the multi-functional roles of *BolANT3* in the future.

The remaining large numbers of candidate upstream regulators identified in our yeast one-hybrid assay in both *Arabidopsis* and cabbage provided promising, diverse, and valuable targets for these translational applications. The rapid technical growth of modern molecular techniques, together with the newly identified candidate breeding target genes, would help speed up the development of more diverse *Brassica* vegetable cultivars with better disease resistance, higher nutritional value, and desired growth phenotypes.

## 4. Materials and Methods

### 4.1. Plant Materials and Growth Conditions

*Arabidopsis thaliana* Col-0 [37], cabbage (*Brassica oleracea* var. *capitata* L.) ‘02–12’ [27], cabbage inbred line QH-10, cabbage double haploid ‘M18-15’, and tobacco (*Nicotiana benthamiana*) were used in this study. For cabbage, seeds were planted in a blend of matrix, vermiculite, and perlite in a 1:1:1 ratio. These were then cultivated in a growth chamber maintained at 25 °C, with a light intensity of 125 μmol·m^−2^·s^−1^ and a light/dark cycle of 16 h light and 8 h darkness. As for the *Arabidopsis* seeds, they were first incubated in darkness at 4 °C for 48 h to ensure synchronized germination. Subsequently, these seeds were also planted in the same 1:1:1 mixture of matrix, vermiculite, and perlite. The *Arabidopsis* seeds were then grown in an incubator set at 22 °C with a light intensity of 125 μmol·m^−2^·s^−1^ and the same 16 h light and 8 h dark cycle.

### 4.2. Gene Cloning and Sequence Analysis

*BolANT1* (*BolC03g073740.2J*), *BolANT2* (*BolC01g001400.2J*), and *BolANT3* (*BolC07g059630.2J*) were retrieved by using the *Brassica* database (BRAD) (http://brassicadb.cn, accessed on 10 December 2021). The PlantCARE online tool (http://bioinformatics.psb.ugent.be/webtools/plantcare/html/, accessed on 10 December 2021) was employed to analyze the cis-acting elements of the *BolCYP83B1* promoter sequences, and they were further visualized using TBtools-II (V2.031) [45]. The amino acid sequence analysis of the BolANTs was performed by using online tools at https://www.ncbi.nlm.nih.gov/Structure/cdd/wrpsb.cgi (accessed on 10 December 2021) and https://www.bioinformatics.nl/cgi-bin/emboss/epestfind (accessed on 10 December 2021). Protein sequences of BolANTs were retrieved from BRAD for *Brassica rapa*, *B. juncea*, and *B. oleracea*, as well as from the *Arabidopsis* Information Resource (TAIR) (https://www.arabidopsis.org/, accessed on 10 December 2021) for *Arabidopsis*. MEGA 11.0 was employed to align the sequences and construct a phylogenetic tree using the neighbor-joining (NJ) method.

### 4.3. Subcellular Localization of BolANT Proteins

The coding sequences of *BolANTs*, devoid of stop codons, were incorporated into the GFP fusion vector, resulting in the construction of pGreen-35S-BolANTs-GFP. To ascertain the subcellular localization of BolANTs, the empty pGreen-35S: GFP vector was introduced into the *Agrobacterium tumefaciens* strain GV3101, which already harbored the pSOUP helper plasmid. When the OD_600_ of the *Agrobacterium* solution reached 0.6, the bacterial suspension was centrifuged at 4000 rpm for 10 min at 4 °C. The resulting precipitates were then resuspended in an infection solution composed of 10 mmol/L MgCl_2_, 10 mmol/L MES (adjusted to pH 5.6), and 100 μmol/L acetosyringone. This suspension was incubated at room temperature for 2 h using a 1 mL syringe, and the bacterial solution was infiltrated into the abaxial surface of tobacco leaves. After two days post-infiltration, the distribution of fluorescent proteins within the cells of the injected tobacco leaves was visualized using Laser Scanning Confocal Microscope (Leica, Wetzlar, Germany).

### 4.4. Yeast One-Hybrid (Y1H) Assay

The Matchmaker^®^ Gold Yeast One-Hybrid Library Screening System (Clontech, Mountain View, CA, USA) was employed for the yeast one-hybrid screen, adhering strictly to the manufacturer’s guidelines. Genomic DNA was isolated from cabbage leaves of variety ‘02–12’ through the CTAB method. The promoter region of *BolCYP83B1*, spanning 2000 bp, was cloned into the pAbAi vector, followed by transformation into the Y1H Gold yeast strain. The successful insertion into the vectors was verified using Matchmaker Insert Check PCR Mix 1 provided by TaKaRa (San Jose, CA, USA). To detect self-activation, aureobasidin A (AbA) was utilized, with a concentration of 150 ng/mL selected for screening purposes. Subsequently, 10 μg of the cabbage library of pGADT7-cDNA was transformed into the yeast strain Y1H Gold-Bait. The yeast cells were then plated onto SD/-Leu/AbA (150 ng/mL) media for screening. After an incubation period of 3 to 4 days, colonies displaying normal growth were identified by colony PCR using the T7 primer (sequence: TAATACGACTCACTATAGGG). Colonies producing PCR bands larger than 500 bp were selected for further sequencing. The sequences of all primers used in this study are detailed in Supplementary Table S4.

### 4.5. Dual-Luciferase Reporter (LUC) Assay

A dual-luciferase assay was utilized to validate the binding of BolANTs to the *BolCYP83B1* promoter. To create the reporter construct, the fragment of the *BolCYP83B1* promoter was inserted into the pGreenII0800-LUC vector, and the CDSs of *BolANTs* were inserted into the pGreenII-62-SK vector to form the effector constructs and subsequently transformed into *Agrobacterium* train GV3101, and a mixture of *A. tumefaciens* carrying the reporter or effector strains was infiltrated into tobacco leaves. Tobacco plants that were infiltrated were subjected to 24 h of dark treatment, followed by 24 h of light exposure. The activity of the promoter was quantified by calculating the ratio of firefly luciferase (LUC) enzyme activity to the internal reference renilla luciferase (REN) using a multifunctional microplate reader (Tecan, Männedorf, Switzerland). The LUC/REN value in the absence of *BolANTs* was established as one. Luciferase activities were measured using the PlantView100 In Vivo Plant Imaging System (BLT, Guangzhou, China).

### 4.6. Genetic Transformation of BolCYP83B1 and BolANT3

The encoding sequence of *BolCYP83B1* was inserted between the *Xba* I and *Kpn* I sites of the pVBG-2307 vector [46], and pVBG2307-*BolCYP83B1* was constructed and expressed in *Arabidopsis* via *Agrobacterium tumefaciens* line GV3101. The transgenic seeds were screened on Murashige and Skoog (MS) medium with kanamycin and identified by PCR. Then, 3-week-oldseedlings of a wild type (WT) and T3 generations of homozygous lines (OE-1 and OE-2) were used for the experiments.

The encoding sequence of *BolANT3* was inserted between the *Xba I* and *Kpn I* sites of the pVBG2307 vector, and the pVBG2307-*BolANT3* vector was constructed and expressed in cabbage ‘M18–15’ via *Agrobacterium tumefaciens* strain GV3101. PCR was used to detect vector insertion. The positive strains of T1 generation were detected by qRT-PCR. *BoACTIN* (*BolC01g044090.2J*) was used as the internal reference gene. Four-week-old unbolted seedlings of the wild type (WT) and T1 generations of homozygous lines (OE-2 and OE-11) were used for the experiments.

### 4.7. VIGS of BolANT3

The 500 bp conserved sequence of the *BolANT3* gene was PCR-amplified and subsequently cloned into the PCVA vector through restriction enzyme digestion and ligation [47]. The recombinant plasmid (designated as PCVA-*BolANT3*) was transformed into *E. coli* DH5α competent cells for propagation. Following plasmid extraction and sequence verification, three experimental constructs, namely an empty PCVA vector, PCVB vector, and recombinant PCVA-*BolANT3* vector, were independently injected into *Agrobacterium tumefaciens* strain GV3101.

For plant transformation, individual *Agrobacterium* cultures harboring distinct plasmids were incubated in LB medium (supplemented with rifampicin, kanamycin, and gentamicin) at 28 °C with 200 rpm agitation until reaching an optical density (OD_600_) of 1.0. The bacterial solution was centrifuged at 6000× *g* for 8 min at 4 °C, followed by two successive washes with equal volumes of infection buffer (10 mM MgCl_2_, 10 mM MES, and 200 μM acetosyringone). After final resuspension in fresh infection buffer, bacterial suspensions were subjected to 3–4 h of dark incubation at room temperature to induce virulence gene expression.

Prior to co-cultivation, equal volumes (1:1 ratio) of PCVB culture suspension were mixed with PCVA-*BoPDS*, empty PCVA, or PCVA-*BolANT3* bacterial suspensions in sterile 50 mL conical tubes. Pre-germinated seeds of cabbage ‘QH-10’ exhibiting approximately 1 cm radicle elongation were immersed in the bacterial suspension mixture and subjected to vacuum infiltration at −0.08 MPa for 40 min. Seedlings were transferred to growth chambers maintained at 22 °C under long-day photoperiod conditions (16 h light/8 h dark). Leaf glucosinolates were extracted 21 days after transplantation.

### 4.8. Total Glucosinolate Extraction and HPLC Analysis

The procedure used for collecting plant leaf samples for GLS analysis mirrored a previously described method [20,48], with necessary adaptations carried out to suit the HPLC platform utilized in this study. Essentially, two to three fully mature leaves were taken from each 3-week-old plant. These leaves were immersed in 1000 µL of 90% (*v*/*v*) methanol. Then, samples were disrupted using a 2.3 mm metal ball in a paint shaker at room temperature, followed by a one-hour incubation period under the same conditions. Next, the tissues were centrifuged at 2500× *g* for 15 min, and the supernatant underwent anion exchange chromatography in 2 mL tubes. Following rinsing with methanol and water, the columns were incubated with 210 µL of sulfatase solution overnight. Allyl glucosinolate was used as the standard compound. The desulfo-GLS was then eluted and analyzed via HPLC following a previously detailed procedure [20,48].

### 4.9. RNA Extraction and qPCR Analysis

Total RNA isolation was performed on distinct cabbage tissues (root, stem, leaf, flower, and silique) using the RNAprep Pure Plant Kit (Tiangen Biotech, Beijing, China). An amount of 2 μg of total RNA from each sample was reverse-transcribed using the HiScript III 1st Strand cDNA Synthesis Kit with an integrated genomic DNA removal system (Vazyme Biotech, Nanjing, China). Quantitative reverse transcription PCR (qRT-PCR) was conducted on a QuantStudio^®^3 Real-Time PCR System (Life Technologies, Carlsbad, CA, USA) with the following parameters: 95 °C for 5 min at initial denaturation, followed by 40 cycles of 95 °C for 10 s and 60 °C for 30 s. Reactions were performed in 25 μL volumes containing Hieff^®^ qPCR SYBR Green Master Mix (Low Rox Plus) (YEASEN Biotechnology, Shanghai, China). The endogenous controls were selected as follows: *BoACTIN* (*BolC01g044090.2J*) for cabbage and *AtACTIN2* (*AT3G18780*) for *Arabidopsis*. Gene expression quantification was performed using the comparative threshold cycle (2^−ΔΔCT^) method.

### 4.10. Statistical Analysis

Statistical analysis was performed using SPSS 23.0 software (IBM Inc., Chicago, IL, USA). Statistical significance was determined using Student’s *t*-test. All data are presented as the means ± SE (standard error). Treatments were considered significantly different at *p* ≤ 0.05.

## 5. Conclusions

The *BolCYP83B1* of the indolic glucosinolate pathway in cabbage was selected and functionally validated. The upstream regulators of *BolCYP83B1* were screened and identified using a yeast one-hybrid assay with *BolANTs* as the top candidates. All three BolANTs were confirmed to be localized in the nucleus, and BolANT1 and *BolANT3* were further confirmed to bind the promoter of *BolCYP83B1* by the point-to-point assay and LUC assay. The overexpression of *BolANT3* in cabbage and the VIGS assay of *BolANT3* consistently supported *BolANT3* as a key positive regulator of indolic glucosinolates in cabbage.

## Data Availability

All the data supporting the findings of this study are available within the paper and in its Supplementary Materials, published online. The plant materials used in this study are available upon request.

## Supplementary Materials

The following supporting information can be downloaded at: https://www.mdpi.com/article/10.3390/ijms26073415/s1.
