# Supplementary figures and images for: BolANT3 Positively Regulates Indolic Glucosinolate Accumulation by Transcriptionally Activating BolCYP83B1 in Cabbage
Source: Int J Mol Sci. 2025 Apr 5;26(7):3415. doi: 10.3390/ijms26073415 (PMC11989275; doi:10.3390/ijms26073415)

## Slide 1
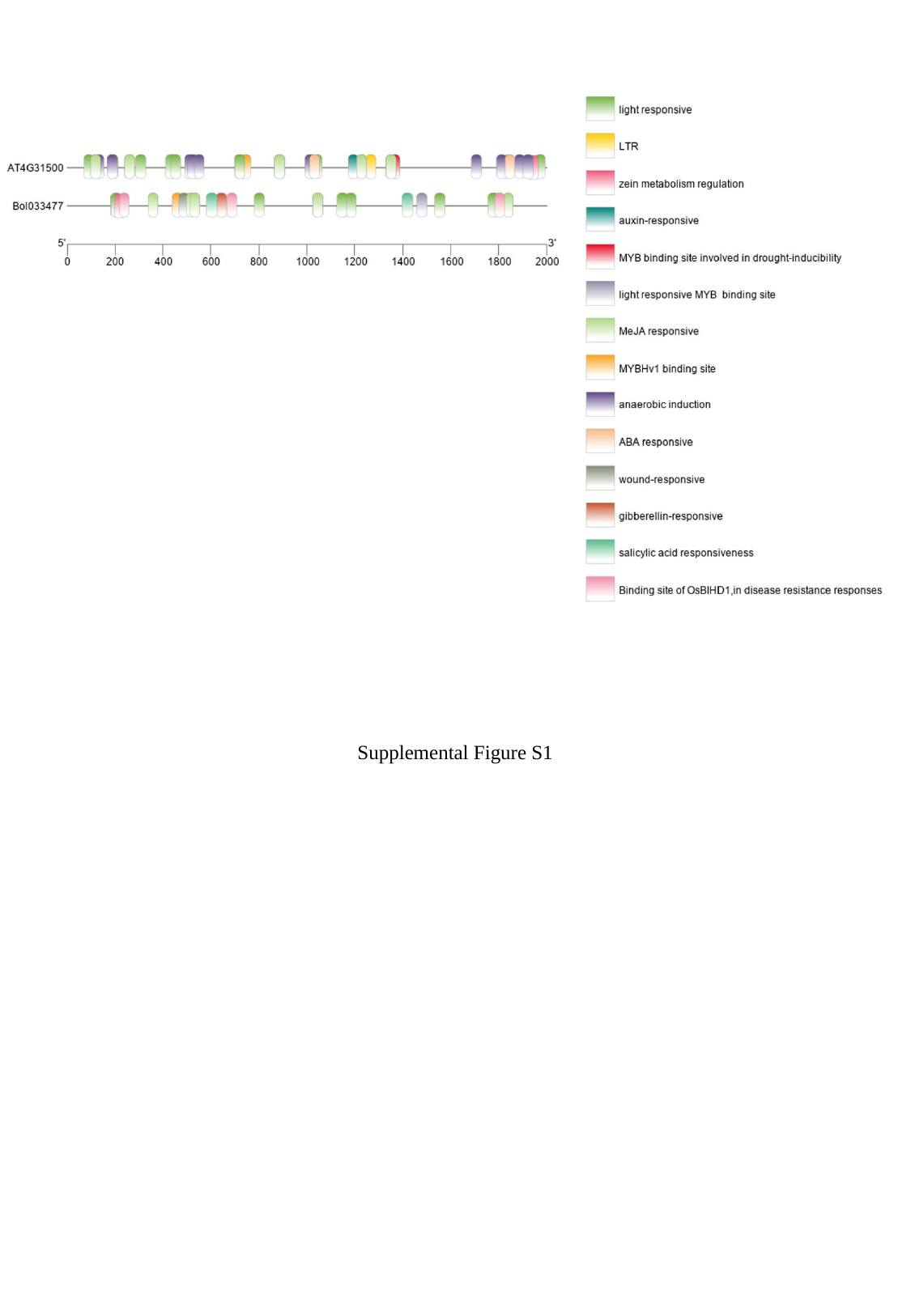

Supplemental Figure S1

## Slide 2
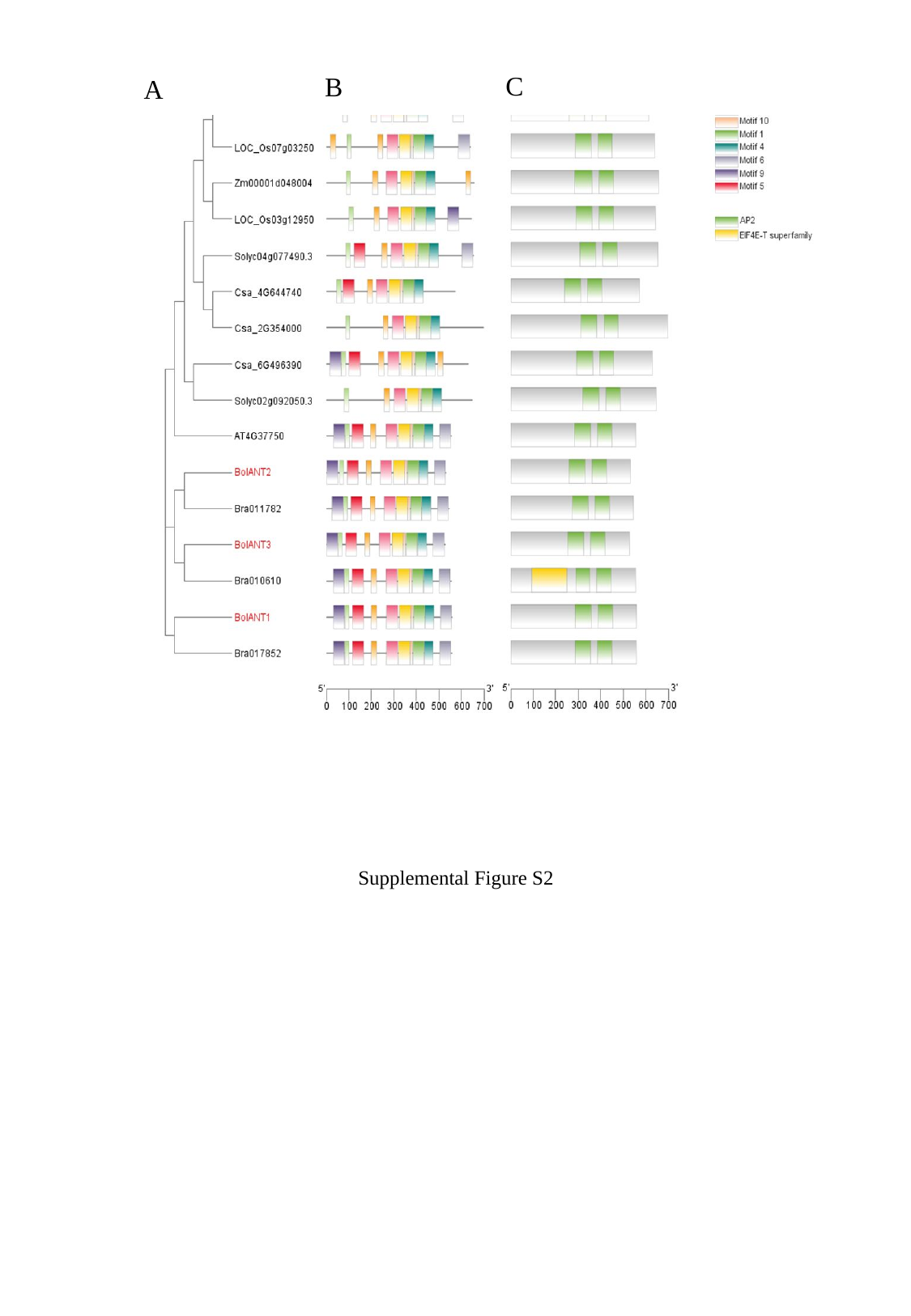

C
B
A
Supplemental Figure S2

Supplement: Supplementary file 1 [file ijms-26-03415-s001.zip › Supplemental Figures S1-S2, 20250216.pptx]
